# Supplementary material for: Effects of Different Drying Methods on Untargeted Phenolic Metabolites, and Antioxidant Activity in Chinese Cabbage (Brassica rapa L. subsp. chinensis) and Nightshade (Solanum retroflexum Dun.)
Source: Molecules. 2020 Mar 13;25(6):1326. doi: 10.3390/molecules25061326 (PMC7145292; doi:10.3390/molecules25061326)
Supplement: Supplementary file 1 [file molecules-25-01326-s001.pdf]

A

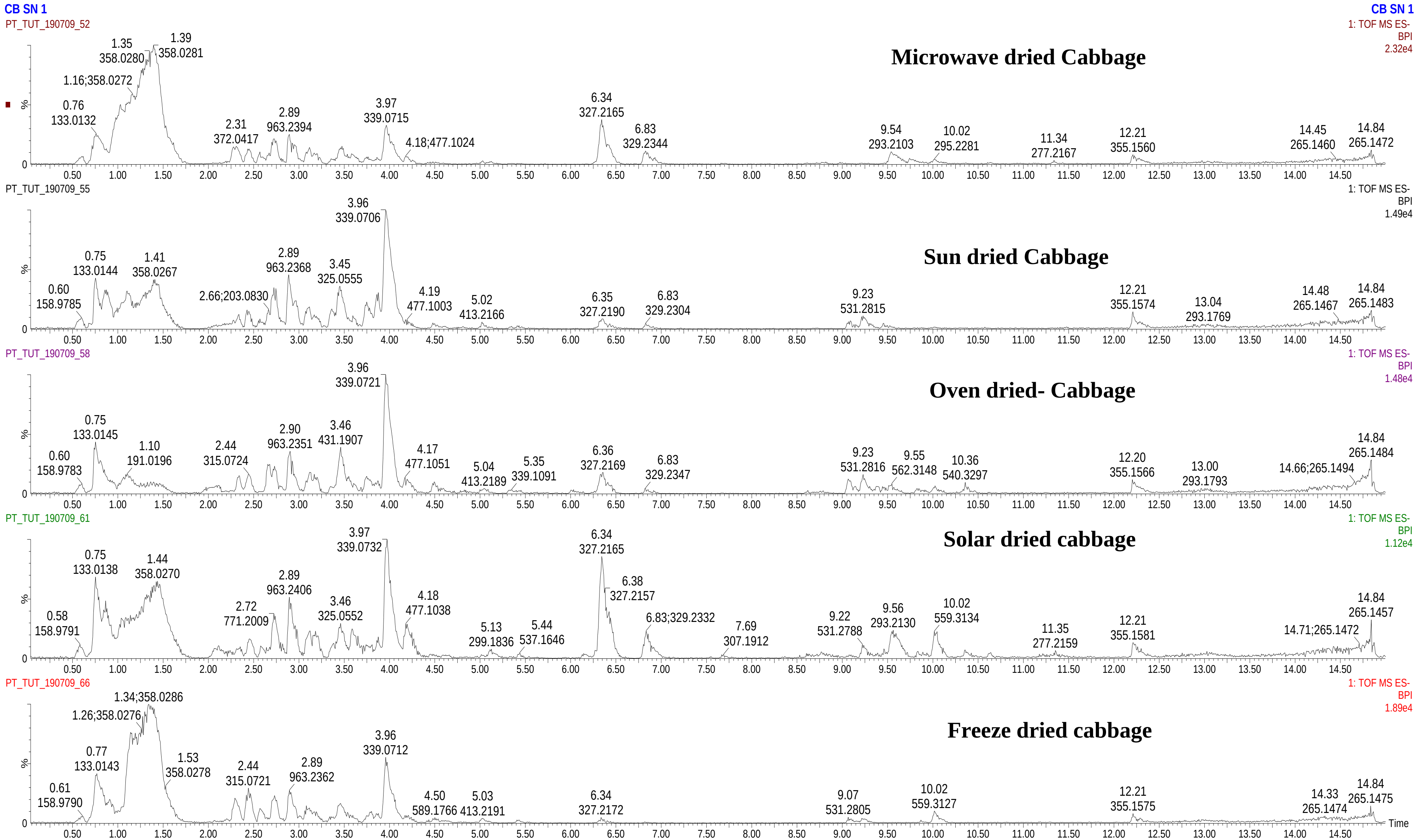
Microwave dried Cabbage
Sun dried Cabbage
Oven dried- Cabbage
Solar dried cabbage
Freeze dried cabbage
CB SN 1
PT_TUT_190709_52
PT_TUT_190709_55
PT_TUT_190709_58
PT_TUT_190709_61
PT_TUT_190709_66
1: TOF MS ES-
BPI
2.32e4
1.49e4
1.48e4
1.12e4
1.89e4
%
Time

B

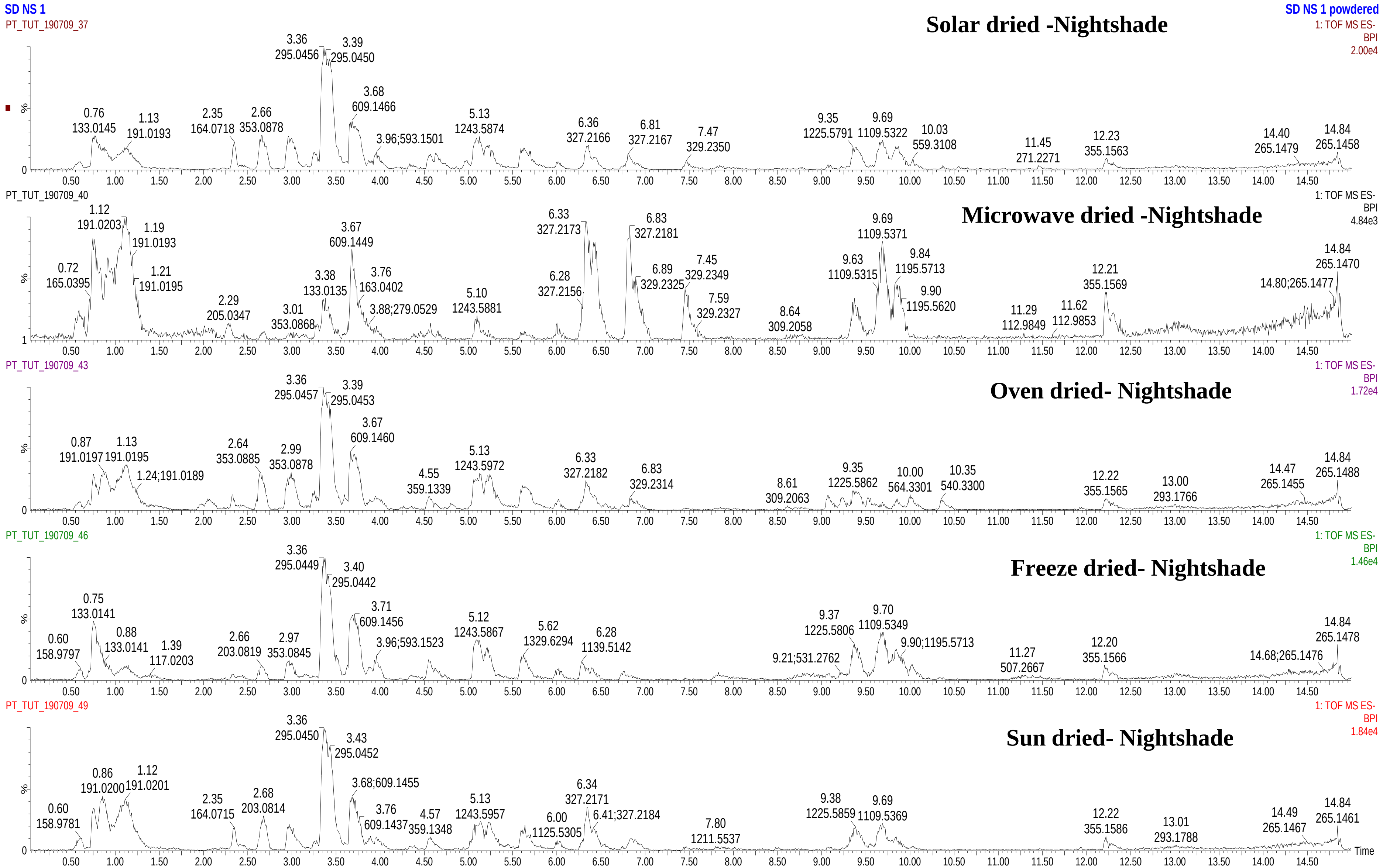

Supplementary Figures 1. Comparison of UPLC–Q-TOF/MS chromatogram illustrating the changes in phenolic compounds in (**A**) Night shade leaves and (**B**) Night shade leaves subjected to different postharvest drying methods. The relative peak intensity is normalized, and peaks are expressed as the percentage highest peak intensity
